# 

**Published:** 1901-06

**Authors:** 


### PERSONALS.

Dr. F. S. Hewitt, of Meshoppen, has moved to West Pittston, Pa.

Dr. Joseph W. Parker, of South St. Joseph, Mo., has been transferred to the Stock Yards Station, Kansas City, Kan., where he will continue his work as Inspector of the Bureau of Animal Industry.

Dr. J. W. Montague, of York, Pa., has entered the Bureau of Animal Industry, and is now stationed at New York City.

Dr. J. G. Rutherford, V.S., graduate Ontario Veterinary College, has been appointed by the Canadian Government as representative to Great Britain in importation of thoroughbred cattle.

Dr. John Dice McLaren, recently of Agricultural Station, South Dakota, now of Cleveland, Ohio, was a visitor to the Pennsylvania State meeting in March.

Dr. J. C. McNeil, of Pittsburg, whose photograph was among the illustrations of the Pittsburg *Sunday Press* of February 24, 1901, is an attache of the Department of Fire.

Dr. John Nott, of Clay Centre, Kan., has been seriously ill, but is now on the way to recovery.

Dr. J. Stewart Lacock, of Allegheny, Pa., escorted a party of about fifteen instructors in the preparatory schools of Allegheny to Philadelphia, in April, where they were the guests of the University of Pennsylvania.

Dr. A. B. Gay, of Randolph, Vt., has met with the misfortune of having his leg taken off below the knee.

Dr. J. E. Spindler, of Tyrone, Pa., has removed to Pittsburg, where he will succeed to the practice of Dr. P. K. Jones, who has gone to West Virginia.

Dr. N. Silverthorn, of New Castle, New South Wales, a graduate of the Ontario Veterinary College of 1884, is the only Canadian veterinary surgeon practising at the Antipodes. Dr. Silverthorn went to New South Wales in 1885, and has built up a splendid practice in that country.

Dr. David Waugh, of Washington, Pa., has just returned after an eventful trip to South Africa with 850 horses from the United States for the British Army in South Africa. A loss of twenty-four on the voyage is all that is recorded on his trip.

Dr. William H. Pendry, of Brooklyn, was an active advocate of the State law in New York placing glanders under the direction and control of veterinarians and removing it from the province of the State Board of Health.

Dr. William B. Werntz has retired from the proprietorship of one of Philadelphia's suburban hostelries, and will make his future home in Philadelphia. After a period of rest he will re-enter upon veterinary practice.

Dr. R. R. Mehaffy, of Wilmington, Del., met with a painful accident in April as a result of one of his equine patients kicking him in the face.

Dr. H. M. Gohn, of St. John's, Michigan, is again in the harness of active practice after a serious experience with septic fever.

Dr. Leonard Pearson, State Veterinarian, was a visitor to Pottsville in April on official work connected with the State Live-stock Sanitary Board.

Dr. John J. Miller, of Sioux City, Iowa, will hereafter be associated with Dr. H. McKillip, of Chicago, in veterinary practice in that city.

The *St. Paul Standard*, of Paterson, N. J., published in the interests of St. Paul's Church, reports the continued improvement of Dr. William Herbert Lowe, president of the Young Men's Association connected with that church.

Dr. F. S. Sheppard, of Gowanda, N. Y., is an admirer of trotters, and finds time to try the mettle of his friends' fast roadsters on the roads around the town.

Dr. E. T. Davison, United States Inspector of the Bureau of Animal Industry, has been transferred from Leavenworth, Kan., to Rushville, Neb.

Dr. Edgar M. Powell, of Oxford, Pa., is an occasional visitor to the "City of Brotherly Love," and always visits the veterinary department of which he is a graduate.

Dr. F. H. Mackie, of Fairhill, Maryland, has taken the hospital of the late Dr. A. W. Clement, of Baltimore, Md.

Dr. L. A. Greiner, of Indianapolis, Ind., is now assisted in his practice by his son, Dr. Joseph M. Greiner, a graduate of the Indianapolis Veterinary College.

Dr. S. D. Larzelere, of Jenkintown, Pa., has been nursing a series of boils and carbuncles.

Dr. H. D. Gill, of the JOURNAL'S editorial staff, still fills the rôle of horseman for this periodical, and behind his famous roadster "Jack" continues to win laurels among the fast ones on New York's Speedway.

Dr. F. F. Shue, of Yorkana, Pa., is a very active veterinarian in all legislative matters of interest to those engaged in the live-stock industry and the welfare of his adopted vocation.

Dr. E. D. Lyon, of Pike, N. Y., spends much time in handling and driving youngsters of the road and track classes.

Dr. Alexander Glass, of Philadelphia, entertained a number of his friends at a planked-shad dinner at the Corinthian Yacht Club on May 13th. Drs. John W. Adams and W. Horace Hoskins were among the invited guests.

Drs. L. A. and E. Merillat, of Chicago, have severed their connection with the McKillip Veterinary Hospital and College, and have entered upon private practice in the "Windy City."

Dr. E. M. Ranck, of Philadelphia, has proven an efficient and successful secretary of the State and local veterinary organizations of Pennsylvania.

Dr. W. B. E. Miller, of Camden, N. J., is carrying an arm in a sling for an acute attack of inflammatory rheumatism, while Dr. William A. Birch, of Philadelphia, is hobbling around for the same cause affecting the knee-joint of one limb. They were recently noted in one of the sale marts exchanging sympathetic reflections.

Dr. H. P. Eves, of Wilmington, Del., has Dr. C. C. Cole now associated with him in his extensive Delaware practice.

Dr. J. C. Shaub, of Lancaster, Pa., was a visitor to Philadelphia, Pa., and New Brunswick, N. J., early in May, making his trip by carriage.

Dr. J. Gibbins, principal veterinary inspector, located at Vancouver, B. C., finds his post of duty an exacting one, and carries him over a large territory constantly.

Dr. J. M. Carter, of Philadelphia, read an interesting paper on "Tumors" at the May meeting of the Keystone Veterinary Medical Association.

